# Valedictory Address

**Published:** 1873-04

**Authors:** W. W. H. Thackston

**Affiliations:** at the Thirty-third Annual Commencement of the Baltimore College of Dental Surgery.


AMERICAN JOURNAL

OF

DENTAL SCIENCE.

Vol. VI. THIRD SERIES--APRIL, 1873. No. 12.

### ARTICLE I.

### *Valedictory Address.*

Delivered by W. W. H. Thackston, M. D. D. D. S., at the Thirty-third Annual Commencement of the Baltimore College of Dental Surgery.

GENTLEMEN OF THE GRADUATING CLASS:

It affords me unaffected pleasure to meet you this evening, to renew my connection with *Alma Mater*, and participate in an occasion to which you have looked forward with so much of interest and solicitude; an occasion for which you have labored and toiled with a diligence and fidelity that has secured you the most complete and gratifying success.

As faithful students, you have had our sympathy in all the difficulties you have encountered and overcome; and now, in this your hour of triumph, we would add our congratulations and good wishes, to increase in some degree the measure of your happiness.

The frosts of thirty winters have touched and thinned our locks since in these halls we sat at the feet and listened to the instruction of the Fathers of Dental Science. We truly and properly designate them "Fathers," for they constituted the first, and then the *only* organized and incorporated faculty of instruction for our specialty the world had seen. We met the labors, experienced the anxieties, and cherished the hopes which, in succession, have tested your strength, disturbed your repose, and exhilarated your spirits ; and at last we rejoice in the success which to-night crowns and accomplishes your own virtuous and laudable ambition.

But we are admonished by the "sere and yellow leaf," by the vacant places, by the absent forms, and stilled voices of our friends and co-laborers, that a great and eventful lapse of time now separates us from all those scenes, pursuits and associations; that we stand alone; the last, the solitary survivor of our class. The "grim Reaper" has spared neither teacher or class-mate; but with ruthless sweep has stricken and gathered them all to his cold embrace.

It is easy, it is pleasing, and yet, in some degree, mournful and sad, to retrospect and live over again the days and years of college experience; to arouse the emotions, to awaken the hopes and anticipations, and to rekindle the fires that burned upon the altar of our hearts.

The ties of friendship then knit and woven endure with the web and woof of life. The labors and hardships, the trials and struggles we then encountered, when viewed through life's later experience, resolve themselves into grateful memories; while the pleasures and enjoyments, the achievements and triumphs, are invested with perennial freshness and verdure.

Whatever positions, young gentlemen, you may be called to fill—whatever destiny, fate or your own future efforts may hold in store for you, believe me, when I assure you, that you will look back to your connection with this institution whose honors you have won, and we trust will worthily bear, as a happy, happy period in your existence. Its friendships will brighten many a dark and gloomy hour; its memories will gild many a towering cloud; its "souvenirs" will quicken many a feeble and fainting pulse.

Fortunate, peculiarly fortunate are you in your circumstances and surroundings; as students, you have sought and enjoyed the training and instruction of the oldest and most renowned Dental School of the world; you have been aided by the large experience, the unlimited resources, the multiplied appliances, and the acknowledged talent of a full corps of instructors, in almost every department of medicine and general surgery, as well as in what constitutes the peculiar features of your own specialty. Your facilities have been full and complete; your range of study has been wide and ample, and you have measured up to all the conditions of graduation as Doctors of Dental Surgery.

It now remains for you to go forth and illustrate in practice what you have so diligently cultivated in theory; to fulfill the hopes and redeem the trust so confidently reposed in you by your friends and instructors.

We know, gentlemen, that upon occasions like the present it is usual to comment upon the incidents and duties of professional life; to picture the difficulties and dangers, the vexations and disappointments you must expect to meet, as well as to portray the brighter scenes and happier circumstances which relieve the burdens of professional toil and give a silver lining to every cloud of despondency or discouragement.

It is also usual that some allusion is made to the great and general principles of *success*, which underlie and control the fortunate results of all the pursuits, vocations and professions of life; and we might adopt *professional excellence* as a suitable theme for discussion and illustration, and trace out the paths by which, in time, you may reach the goal of your highest aspirations. We might tell you that in severing your connection with this college, you should become none the less diligent students; that your application and research, so far from abatement, should now be more earnest —should take a wider range and broader scope; that you should not only keep abreast with the progress and improvements in your department, but lend your own assistance in exploring the yet untrodden fields, and in solving the yet hidden problems of Dental Science.

With warning finger we might point out to you the rocks and shoals on which so many have been wrecked; the quick-sands, sloughs and whirlpools, in which so many who gave promise of a brilliant career have foundered, gone down, and disappeared from mortal vision and mortal hope.

We might read you an essay upon elevated character; upon stern and inflexible integrity; upon unbending firmness of purpose; upon the graces, the virtues and the accomplishments which should distinguish the dentist in all his relations, social and professional.

But we have thought these objects might be as well accomplished, and the occasion more profitably improved, by asking your consideration of a character identified for the last thirty or more years with the educational interests of Dental Science, and which afforded in life a most striking illustration of all the qualities and attributes which distinguish a great and good man.

An all-wise and a mysterious Providence has visited us with a bereavement, in which all who love truth, who honor virtue, who admire genius, and who worship piety, must sympathize. We look in vain to-night for a well remembered face; we listen in vain for a familiar voice, whose words of cheer, of encouragement, and of parting instruction, so often charmed our ears and impressed our hearts. The bright and sparkling eye that beamed and melted in looks of love and tenderness, has lost its light. The eloquent voice that thrilled your feelings, that aroused your energies, that directed your labors, that threw the magic of an enchanter's wand over the most difficult problems of science, and invested the dry details of technical study with the interest and charm of a splendid epic, is now hushed in the stillness of the grave. We see around us, entwined with the garlands of triumph, the emblems of grief; with the laurels of victory, the cypress of mourning.

Professor Thomas E. Bond, President of the Faculty, and the last surviving founder of this school, is now numbered with our illustrious dead. And we come to-night not only to lay our offering and tribute of affection upon his tomb, but to improve this last hour of your college life by setting before you the character and example of one so long, so earnestly, and so efficiently identified and associated with this institution. And not only with this particular school, but with all the agencies and measures which for a long series of years have been inaugurated for the cultivation and development of Dental Science, and its establishment and recognition as one of the "Liberal Professions."

"*De mortuis nil nisi bonum*" is a maxim prescribed and dictated by charity, as well as affection; but our subject to-night makes no demand upon our forbearance; no requisition upon the "Lethean waters," but justice and truth will tax and strain our powers, to place fairly before you the character of one who, in life, was a bright and conspicuous exemplar of all the qualities and all the virtues that adorn, dignify and ennoble humanity.

Happily, our task imposes no defence of human frailties, no palliation of human follies, no veiling of moral or intellectual deformities; but the simple exhibition of a character pure in its elements, grand and symmetrical in its development, and in its proportions.

We do not mean to say that our departed friend was absolutely faultless; that no taint of the fall, no human imperfection, clouded the brilliancy of his character or abated the measure of his usefulness. Dr. Bond was, after all, but a man, with the inherent and entailed imperfections which distinguish man from the "higher intelligences." But we do mean to say, that through labor, through discipline and through grace, his imperfections were either purged away, or they were eclipsed and obscured by the lustre of his shining virtues.

The loss of such a man is in every sense a calamity; it is a bereavement which not only family and friends must recognize, but which society acknowledges; which civilization deplores, and which *science* and *letters* must lament as irreparable. A great and good man has fallen; a bright and shining light has gone out: you, gentlemen, have listened to his last words of counsel and instruction; but in dying he has bequeathed you the rich legacy of his great and almost life-long labor in your behalf, and an example of devotion to truth, and fidelity to principle—of unfaltering industry and energy in every good word and work—that you may study with profit and imitate with incalculable benefit.

Concerning the early life of Dr. Bond, our information is meagre and unsatisfactory. We were for a time under the impression that he was descended from one of the "old Maryland families;" that he was "to the manor born;" but we have recently seen from the public journals that he was a native of the State of New York—where, we suppose, his family resided; where his earlier years were passed, and perhaps where his academic and collegiate studies were prosecuted. Of the events and incidents of that period of his life, we know but little; doubtless, much of interest and much that would be valuable and instructive, could be gathered from the early life of such a man. But it matters not where he was born and reared, or what events or circumstances shaped or controlled his youth and dawning manhood—this much is certain, if there be truth in the maxim, that "the child is father to the man," Dr. Bond gave early promise of a life of honor, usefulness and distinction. He could have been nothing less than what is denominated a "bright boy." He could have been nothing less than a faithful, energetic and devotedly studious young man; for at the age of about thirty, when our acquaintance with him occurred, we found him a ripe scholar in all the departments of college and university instruction; and his mind stored with a fund of knowledge, which embraced and comprised nearly all the subjects of human thought and investigation. He had evidently been trained to systematic study, to searching analysis; and to independent and original thought in the solution of all the questions which engaged his attention. Could we pass in review before you his youth and early manhood, lessons of wisdom and examples of virtue might be gathered all along the straight and narrow path which he followed, up to the full development of his extraordinary powers, and to the perfect and complete maturity of his master mind.

Having adopted medicine as the profession of his choice, he matriculated in the medical department of the University of Maryland, and here the sympathies of his heart, the energies of his nature, and the disciplined powers of his mind, found full and active play; and it was no source of wonder, or cause of surprise, that he achieved distinction as a medical student, and graduated with the highest honors of his school.

Having received his degree, Dr. Bond determined to make the city of Baltimore the field of his practice, and we hazard little in saying that few physicians have commenced a career under more auspicious circumstances; few, we presume, have entered a field of richer promise, or engaged advantages of equal value. His honored father, a popular and distinguished physician of this city, had retired from a large and lucrative practice. His mantle descended upon a son, who wore it worthily, and kept its ermine pure and spotless. Possessing the confidence of the people, master of all the resources of his art, there was nothing before him but a prospect of brilliant success, leading to fortune and professional distinction. And had he listened to the promptings of mere worldly ambition, to the suggestions of avarice, or yielded to the greed of gain, he would have lived and died simply a successful, a distinguished, and a wealthy practitioner of medicine.

But Dr. Bond was endowed with a great, warm and sympathetic heart, as well as with a brilliant and cultivated intellect; he readily perceived, and very naturally became interested in the wants and needs of society, outside his profession proper; and it was more than his nature could brook or bear, to be conscious of a public necessity without giving his thought and effort to the relief of that necessity. From the same mental and moral characteristics he was exceeding jealous and watchful of the rights and interests of society. He guarded with vigilant care the portals of public intelligence, of public honesty and virtue; if either were threatened or assailed, he gave the signal note of warning, and fearlessly threw himself into the breach. Against all pretenders and quacks in science, against fanatics and heretics in religion, against demagogues in politics, and against all knaves and impostors in society, he firmly and persistently arrayed himself.

In the lecture room, on the rostrum, through the secular and religious press, few men of his day did more to instruct the public, to teach science, to defend and establish truth, and to expose and denounce evil and errors, than Thomas E. Bond.

And here let me pause to say that our friend was no knight errant of the Cervantes school, going about seeking tilts and adventures. True, he *was* a knight; a knight "without fear and without reproach;" but his was the chivalry of the enlightened Christian; his "tilts and tourneys" were in obedience to an enlightened judgment, and illustrated a higher civilization; were responses to the demands of duty and the requirements of a wholesome state of society. It will be remembered by many who hear me to-night, how often in this city, after battling with the hydra of disease in his practice as a physician, he has hurried away to battle with the hydra of error and imposture.

While for the whole pack and brood of charlatans and pretenders, who live and fatten upon the ignorance and credulity of men, Dr. Bond cherished an utter and uncompromising disgust and contempt, and while for such he always kept a "rod in pickle," there was nothing of malicious gratification, nothing of personal satisfaction in the terrible flayings and castigations to which he submitted them, beyond the consciousness of having discharged a sacred duty to society.. But these diversions and exercises, however enjoyable and beneficial to the public, did not promote his interests or enhance his prospects as a physician. But heavy as were his blows, and keen and smarting as were his thrusts, he never sacrificed his benevolence or compromised his dignity as a Christian gentleman.

In early manhood Dr. Bond became the subject of religious impressions and convictions, which resulted in his connection with one of the most prominent of the Protestant denominations, and in this new relation another wide field was opened for the exercise of those rich and peculiar powers of mind and heart with which he was so happily endowed. With the zeal and fidelity which distinguished him in the other pursuits and relations of life, he met and performed his Christian duties. With stores of knowledge, with a wealth of learning, that placed him in such striking contrast with his fellow men, he was yet an humble and devout, as well as a most active and laborious follower of his Master. No arrogance, or vanity, or egotism, dimmed the lustre or impaired the symmetry of his Christian character. As a servant, he worked in his Master's vineyard; as a faithful steward, he improved the talents committed to him.

This church relation speedily assumed an interest and importance that drew largely upon the time and thought of our friend; it so engrossed his attention and taxed his energies that the demands of an extensive medical practice could not be fully met—and from about this period Dr. Bond ceased to regard the practice of medicine as the leading pursuit of his life. Though always ready to answer the appeals of the sick and suffering, he did not court practice for pecuniary reward, or personal distinction—but devoted himself to the benevolent but less remunerative mission of teaching and instruction in the lecture room, through the press, and in the sacred desk.

We cannot, in the limits of such an address, do justice to the character of Dr. Bond as a preacher of the Gospel, or as a religious journalist; but must content ourself with a few brief observations upon the status he maintained in this dual relation to his church and the public. We have told you that he was a man of quick, clear and sharp perception, of great and varied scholarly attainments, a close and accurate observer. Few events of interest escaped his attention; and few subjects of importance resisted his power of analysis. Added to these qualities, he possessed that wonderful attribute which is denominated "genius." With pleasing address and polished manners, with genial humor and sparkling wit, and with a readiness which, under all circumstances, commanded his affluence of ideas and of words, Dr. Bond as a speaker, as a lecturer, and as a preacher always measured up to the requirements of the occasion. He was clear, forcible and impressive, without the advantage of a commanding person, or the charm of a musical voice; he always commanded the attention of the most critical and appreciative audiences, and rarely, if ever, failed to repay that attention, with such a presentation of his subject, as was both entertaining and instructive.

But it was, perhaps, as an editor, as a religious "journalist and reviewer," that he achieved his highest distinction, and won his most imperishable trophies. As a writer, his scholarship, his store of knowledge, his versatile talents, and his ease and happiness of expression, would have made him a man of note in any field of letters, or in any department of science; and when applied to the advocacy, the illustration or the defence of his religious faith, placed him at once in the fore-front, and along side the ablest religious journalists the present age has produced. The announcement of his name as editor or co-editor of a religious journal always attracted the attention and secured the patronage of the public. This prompt recognition of his merits by all classes and denominations, was part of the tribute men ever pay to "genius," and that genius illuminated the columns of his journal, and threw around his contributions a fascination that few could resist. Always ready, always poised, he could at will charm your fancy, enlighten your understanding, or convince your judgment.

But the most prominent feature in Dr. Bond's journalism was the strong and thorough infusion of "common sense" which pervaded his editorials and contributions to the press. We do not remember ever to have seen an article from his pen that even an enemy would have pronounced absurd or foolish; for what he wrote was pith and point and purpose—garnished with wit that sparkled, and sometimes spiced with sarcasm that stung and blistered. His argument always strong, his invective was terrible; and the scathings and scorchings that evil-doers provoked at his hands were such as few could withstand or endure.

Of all the traits in Dr. Bond's well marked organization, none, perhaps, were more conspicuous than his *positiveness*. He was in no sense a negative man; he did not listlessly or apathetically drift into the current of events; he did not float upon the tide of popular fancy or popular fashion, but was fearlessly pronounced in all his relations and positions. Having investigated a proposition, having apprehended the line of duty, he pursued that line with Spartan courage and Spartan constancy—looking alone to the reward of a good conscience, and to the approval of that Master whose name he daily honored.

As illustrative of the decision and firmness to which we have alluded, we may here mention that, thirty or thirty-five years ago, realizing a public necessity, and in obedience to a conviction of duty, Dr. Bond associated himself with a few kindred spirits in the effort to rescue a valuable department of science from prostitution and debauchery, and to give to it the dignity and character of a "Liberal Profession." In this work, apparently so suicidal to his interests and prospects of preferment, we had reason to believe that he was strenuously opposed by his friends and advisers, who not only brought the weight of argument, but the shafts of ridicule, to bear upon what they called his quixotic purpose of making a respectable or recognized science from an art which, with a few notable exceptions, had up to that period been regarded as the undisputed domain of the empiric and charlatan. But, true to his convictions of right, and apprehending the value of the results to be achieved, he shook off or turned aside all opposition, and with the lamented Harris, Hayden and others, organized this college, and accepted one of its most important chairs.

As we have elsewhere said,* "having put his hands to the plough, he looked not back," but with unfaltering constancy and fidelity to Dental Science, he watched by its cradle, and, nursing its growth and development, relinquished his duties and ceased his labors in its behalf with his last expiring breath.

Without expecting any approach to adequate compensation, without a reasonable hope that he would live to see the college he had aided in founding, either endowed with a supporting fund or made self-sustaining by sufficient patronage, Dr. Bond at once set himself industriously to work and prepared a full and complete course of lectures, meeting exactly the wants of the dental student. These lectures were always kept fully up with the progress of science; and all the improvements and discoveries of the day were carefully noted, and thoroughly discussed and investigated in the pleasing and impressive style that marked all his public exercises and performances.

As a performer, Dr. Bond not only fulfilled all the requirements and responsibilities of his position as a teacher, but maintained with his classes the relation of a warm and sympathizing friend. Easy and affable in his manners, genial in his feelings and disposition, his students found no difficulty in approaching him, and experienced no restraint or embarrassment in seeking his counsel and assistance in any matter affecting their interest or their happiness; and we here venture the assertion, that no pupil ever sought his

*Memorial in Virginia Dental Association.

advice or appealed to his assistance in vain. Cordially entering into their feelings, he would realize their wants and needs, and cheerfully afford all the relief in his power. In his intercourse with his students, his social virtues were no less conspicuous than his scholarly attainments and professional ability. In the chair or out of it, in the lecture hall or in the social circle, he was in every sense a "model man."

To promote the interests of dental science, to benefit the profession at large, as well as to aid the progress of his students, Dr. Bond prepared and published an able work in his department, which has been accepted and adopted as a Text Book in all the colleges, and as an indispensable acquisition to all professional libraries. Recognizing his peculiar qualification, to Dr. Bond was entrusted the composition and authorship of the "College Diploma," and the Latin parchment for which you have so wrought and struggled, and which at last you have fairly won, attests the purity of his taste and the accuracy of his scholarship.

To the professional journals he was a generous and valuable contributor. Amid the activities of his busy life, amid the press upon his time and thought, he found opportunity through all the avenues of knowledge to foster and encourage the steady growth and improvement of dental science. But his thinking and writing and speaking are over. In his own last words, "his work is done." And in reference to him, we cannot escape the suggestions of the text, "Blessed are the dead who die in the Lord, for their works do follow them." Dr. Bond so lived and so died that his works will live after him ; "will follow him."

And now, gentlemen, in conclusion, let me commend to you the love of truth and virtue, the firmness of purpose, and the devotion to principle, which so distinguished the late President of this college as the most reliable safe-guard against failure and misfortune, as the straightest pathway to honorable position and universal esteem. Let me impress upon you his example of tireless energy, of patience and perseverance, and of perfect fidelity to every duty.

Live as he lived, and you will achieve a success which should abundantly satisfy your highest aspirations. Live as he lived, die as he has died, and "your works will follow you."

---

### ARTICLE II.

### *Is Woman Adapted to the Dental Profession?*

